# Analysis of long-term outcomes in 44 patients following pelvic exenteration due to cervical cancer

**DOI:** 10.1186/s12957-020-01997-3

**Published:** 2020-09-02

**Authors:** Agnieszka Lewandowska, Sebastian Szubert, Krzysztof Koper, Agnieszka Koper, Grzegorz Cwynar, Lukasz Wicherek

**Affiliations:** 1 2nd Department of Obstetrics and Gynecology, Centre of Postgraduate Medical Education, Bielanski Hospital, Ceglowska 80 St, 01-809 Warsaw, Poland; 2 Clinical Department of Gynecological Oncology, The Franciszek Lukaszczyk Oncological Center, Bydgoszcz, Poland; 3 Department of Chemotherapy, The Franciszek Lukaszczyk Oncological Center, Bydgoszcz, Poland

**Keywords:** Pelvic exenteration, Cervical cancer, Cancer recurrence, Vaginal fistula

## Abstract

**Background:**

Pelvic exenteration (PE) may be associated with prolonged overall survival (OS) in selected patients with advanced or recurrent cervical cancer. However, the factors related to improved survival following PE are not clearly defined. The aim of this study was to perform a retrospective analysis of OS rates in a group of patients undergoing PE in order to identify the factors related to improved long-term outcomes.

**Methods:**

Our study group consisted of 44 patients, including 21 squamous cell cancer (SCC) patients, 22 patients with adenocarcinomas (AC) of the cervix, and one patient with undifferentiated cervical carcinoma. The patients were categorized according to the type of surgery, namely, primary surgery (12 patients) or surgery due to cancer recurrence (32 patients).

**Results:**

In the group of patients with recurrent cervical cancer, we found that improved OS correlated with the SCC histological type and the presence of vaginal fistula. The need for reoperation within 30 days and the presence of severe adverse events significantly worsened the prognosis. We found a non significant trend toward improved survival in those patients with tumor-free margins. Lymph node metastases, the initial stage of the disease, the time to recurrence, and a history of hysterectomy had no impact on patients’ OS. In the group of patients undergoing primary PE, we observed a trend toward improved survival among those diagnosed with vaginal fistula.

**Conclusions:**

Pelvic exenteration seemed to improve the long-term outcomes for patients with SCC cancer recurrence and vaginal fistula whose surgery was unrelated to severe adverse events.

## Introduction

Pelvic exenteration was first described by Brunschwig in 1948 as a palliative procedure for patients with advanced pelvic malignancies [1]. Since then pelvic exenteration has become a routine surgical procedure—an option of choice for treating the relapse of pelvic malignancy due to either palliative or therapeutic indications for surgery—and has resulted in long-term outcome benefits for patients. However, despite improvements in surgical techniques and better postoperative management over the last two decades, such an extensive and complex surgical procedure is still associated with a high rate of postoperative complications. Nevertheless, the survival benefit of the procedure has been well documented, particularly in cases of cervical cancer relapse where negative resection (R0) margins can be achieved [2, 3]. Achieving an R0 resection is possible in more than 70% of cases and remains one of the most important determinants of survival benefit [4–7]. For this reason, PE is becoming a therapeutic option for an increasing number of cases of pelvic malignancy recurrence.

The promising results of the application of exenteration in treating the recurrence of rectal cancer have also revealed that one of the most difficult tasks for surgeons during the operation is to distinguish properly between the tumor nest and radiation-induced fibrosis or local inflammation [8]. The majority of exenterated patients with gynecological malignancies are operated on due to cervical cancer relapse and so have a history of pelvic chemoradiation. As in the case of rectal cancer, proper determination of the border between cancerous and healthy tissue within the radiated field (pelvis) is important. Hockel et al. have proposed the idea of performing laterally extended endopelvic resections (LEER) [9]. The application of such an ultra-radical type of surgery would allow for a better designation of the range of the neoplasm invasion. Moreover, the laterally extended surgical technique would enable the achievement of negative margins in cases of both central pelvic recurrence and pelvic side wall recurrence. Additionally, Hockel et al. have demonstrated promising results for both locally invasive and relapsed gynecological malignancies in cases where pelvic exenteration is part of the therapy [9].

Although PE remains the only curative option for patients with cervical cancer pelvic recurrence, the impairment in patient quality of life and the high risk of adverse surgical events have generated much concern over the qualification for PE surgery. One of the most important factors in determining whether a patient qualifies for the surgery is the correlation between cervical cancer relapse and the appearance of clinical symptoms. These symptoms include chronic pain, problems voiding or defecating, and the formation of vaginal fistulas; the latter in particular has been shown to have a strong negative impact on the patient’s quality of life [10]. The decision to perform such an extensive surgery seems easier in the case of the appearance of clinical symptoms, as neither palliative radiotherapy nor chemotherapy is possible. The decision about a patient’s qualification for PE should be based on an analysis of prognostic factors to assess the potential for long-term outcome. However, PE is not performed frequently; thus, there is a lack of studies to provide data for accurate risk stratification. Accordingly, the aim of the current study was to perform a retrospective analysis of the OS rates in a group of patients undergoing PE due to cervical cancer in order to identify the factors related to improved long-term outcomes.

## Materials and methods

### Study population

We conducted a retrospective analysis of patients (*n* = 44) who underwent pelvic exenteration due to cervical cancer in the Clinical Division of Gynecological Oncology of the Franciszek Lukaszczyk Oncological Center in Bydgoszcz, Poland, from 2010 through 2018. The patients were categorized according to the type of surgery, namely, primary surgery (12 patients) or surgery due to cancer recurrence (32 patients). Patients with recurrence were primarily treated by radical hysterectomy followed by chemoradiation or only by radical chemoradiation (depending on the FIGO stage). In cases of the recurrence treatment with exenteration was provided as an alternative to palliative chemoradiation. The second group consisted of patients treated primarily by exenteration due to a locally advanced disease (FIGO III and IV) and demonstrated associated symptoms like vaginal bleeding, hemorrhage, recto-vaginal, or vesico-vaginal fistula. Those patients also underwent adjuvant chemoradiation. All of the patients included in this study were in good general condition without severe comorbidities (0-2 points in Eastern Cooperative Oncology Group (ECOG) Performance scale). We did not use any specific exclusion criteria. In patients treated due to cervical cancer recurrence, we included only patients whose image studies suggested the possibility of complete (R0) resection. We did not used time to recurrence as exclusion criteria.

All of the patients remained in the follow-up. During the first 3 years following the surgery, the patients were examined by an experienced gynecological oncologist every 3 months, thereafter, every 6 months. The patients underwent physical examination, the imaging studies were performed when necessary. In all cases of a patient’s death, the exact date of death was obtained.

All of the patients included in this study (both with primary disease and cancer recurrence) had a diagnosis confirmed in a final histopathological examination.

### Surgical technique

We included only those patients who underwent one of the following: (1) anterior pelvic exenteration (the removal of the bladder, partial or total resection of the vagina, and removal of the uterus or the vaginal vault); (2) posterior pelvic exenteration (removal of the rectum with or without resection of the anus, partial or total resection of the vagina, and removal of the uterus or the vaginal vault); or (3) total pelvic exenteration (the removal of the bladder, partial or total resection of the vagina, removal of the uterus or the vaginal vault, and the removal of the rectum with or without the anus). In those patients who had already had a hysterectomy, the vaginal vault was surgically removed.

Patients underwent a longitudinal laparotomy extending from the pubic bone to the level above the umbilicus and bilateral/unilateral salpingoophorectomy. In the case of total pelvic exenteration, the pelvis was covered by the omental flap. Pelvic lymphadenectomy with or without para-aortic lymphadenectomy was performed in every case where the lymph nodes had not been removed during the primary surgery. All surgeries were performed by accredited gynecological oncologists (in most cases, L.W.). In each case, preoperative bowel preparation with a mechanical bowel and preoperative enema was administered. All patients received an intravenous antibiotic prophylaxis composed of first-generation cephalosporin, metronidazole, and gentamicin. The majority of the patients who underwent extensive surgery received postoperative parenteral nutrition. The administration of transfusions of red blood cell concentrates (RCC) depended on the patient’s clinical performance; however, most of the patients with postoperative hemoglobin concentrations below 8 d/dL received RCC. We recorded serious perioperative (occurring within 30 days of the procedure) morbidity defined as severe (requiring prolongation of the hospital stay) surgical site infection, the need for reoperation, anastomotic leakage, Bricker neobladder leakage, development of a fistula, ileus that required surgery, or patient death.

For each of the collected specimens, a final histopathological diagnosis was made and an evaluation of free margins and lymph node involvement was conducted.

### Statistical analysis

The categorical data was compared applying Fisher exact test using GraphPad Instat 3.06. The distribution of FIGO stages between squamous cell carcinoma and cervical adenocarcinoma was compared using Fisher exact test with the Freeman-Halton extension (VassarStats; http://vassarstats.net/fisher2x3.html). The groups were then categorized as follows: IB, IIA, and IIB vs IIIA; and IIIB vs IVA and IVB. Survival analyses were conducted using Kaplan-Meier survival curves and the differences in patient survival were compared using the log-rank test. The median survival time was calculated as the smallest time at which the survival probability drops to 0.5 (50%) or below. If the survival curve did not drop to 0.5 or below then the median time could not be computed (was not reached). Median survival and interquartile range (IQR) in particular study groups were calculated using MedCalc 11.4.2.0. The data is presented in Tables 2 and 3. Multivariate survival analysis was conducted using Cox proportional-hazards regression with the stepwise entering method.

In all of the statistical evaluations, the *P* value of < 0.05 was considered significant. Similarly, when the multivariate survival analysis was performed, the model included only variables with *P* value below 0.05.

## Results

### Patient characteristics

We identified 44 patients who underwent total (24 patients), anterior (15 patients), or posterior (5 patients) pelvic exenteration. Forty of these patients had been previously treated with primary chemoradiation while 3 had had a radical hysterectomy followed by pelvic radiotherapy. Thirty-two patients underwent PE due to cervical cancer recurrence, while 12 had primary exenteration at the beginning of their cancer treatment. Patients who underwent primary exenteration had the following indications for surgical treatment: massive hemorrhage (4patients); ileus (3); huge adnexal tumor (1); vesico-vaginal fistula (3); recto-vaginal fistula (1); large tumor with no possibility of radical radiotherapy (2). Nine patients (7 with squamous cell cancer cervical cancer and 2 with cervical adenocarcinoma) were diagnosed with cancer recurrence with either vesico- or recto-vaginal fistula. Eleven patients (25%) experienced severe perioperative morbidity. Table 1 presents detailed patient characteristics.

**Table 1:** Patient characteristics according to the histopathological type of the cervical cancer and type of pelvic exenteration

	Type of the pelvic exenteration			FIGO stages	Previous hysterectomy	Reoperation within 30 days	Tumor cells on specimen margins	Lymph node metastases	Utero-vaginal or intestino-vaginal fistula	Severe morbidity (*n*)
	Anterior	Total	Posterior							
Squamous cell cervical cancer (*n* = 17)	3	12	2	IB – 3; IIA – 0; IIB – 5, IIIA – 0, IIIB – 5, IVA – 3, IVB - 1	2	1	1	1	4	Intestinal fistula (1), reoperation (1), pelvic abscess (2)
Cervical adenocarcinoma (*n* = 14)	3	9	2	IB – 1; IIA – 0; IIB – 4, IIIA – 1 IIIB – 3, IVA – 5, IVB - 0	1	4	4	5	0	Reoperation (4), intestinal anastomotic leakage (1), Bricker neobladder leakage (1), abdominal wall abscess (1)
Undifferentiated cervical cancer (*n* = 1)	0	1	0	IIIB - 1	0	1	0	0	0	Intestinal anastomotic leakage (1), reoperation (1)
Total (*n* = 32)	6	22	4		3	6	5	6	4	13
Pelvic exenteration due to cervical cancer recurrence										
Squamous cell cervical cancer (*n* = 4)	3	1	0	IIB – 1, IVA – 3,	0	0	0	1	3	Intestinal fistula (1), abdominal wall abscess (2)
Cervical adenocarcinoma (*n* = 8)	6	1	1	IIB – 2, IIIB – 2, IVA – 3, IVB - 1	0	0	2	3	2	Reoperation (1), abdominal wall abscess (1)
Total (*n* = 50)	9	2	1		0	0	2	4	5	5

### The survival analysis of patients treated due to cervical cancer recurrence

We found that the initial FIGO stage of the disease had no impact on patient survival following PE (*P* = 0.70). Furthermore, there was no correlation between patient survival and the type of PE (anterior vs total vs posterior; *P* = 0.88) performed. However, we did observe significantly improved OS following PE surgery in those patients diagnosed with squamous cell cervical (SCC) cancer compared with the cervical adenocarcinoma (AC) patients (*P* = 0.01). In the group of SCC patients with cancer recurrence, as many as 1/3 lived longer than 3 years, and 1/4 survived longer than 4.5 years after PE. Among eight patients who are still alive, 4 patients are free from the disease and remain in good condition. Four patients experienced the recurrence of the disease (after 6, 7, 14, and 20 months); three of them received the second line of chemotherapy and they remain stable and disease tumor free. One of these patients was not classified for chemotherapy and she received palliative care.

The presence of vaginal fistula during cancer recurrence was also associated with significantly prolonged survival (*P* = 0.02). However, the need for reoperation significantly worsened the prognosis (*P* = 0. 02), and 11 patients (34%) experienced one or more serious adverse events. Severe postoperative adverse events were associated with poor long-term outcomes (*P* = 0.03). Table 1 summarizes the incidence of adverse events based on the type of PE.

Patients with positive margins had shortened overall survival; however, the difference did not reach the level of significance (*P* = 0.09). Similarly, the incidence of lymph node metastases did not influence patient survival (*P* = 0.44).

**Fig. 1:**
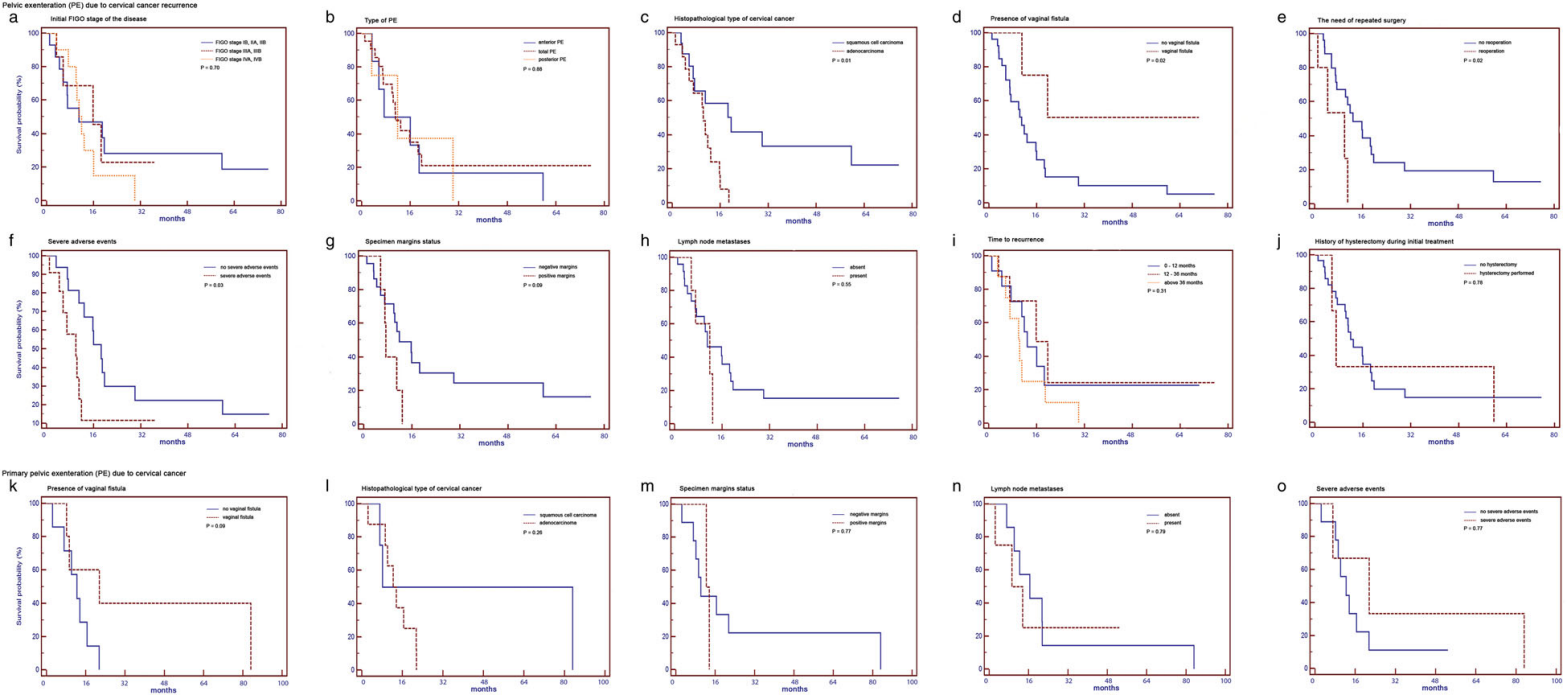
Survival analyses after pelvic exenteration (PE) due to cervical cancer. Survival analysis of cervical cancer patient treated with PE due to cancer recurrence according to (**a**) initial FIGO stage IB, IIA, and IIB (*n* = 13) median overall survival (mOS) = 11.4 months, (interquartile range: 5.5-41.4) versus IIIA and IIIB (*n* = 10) mOS = 16.4 months (3.8-17.1) versus IVA and IVB (*n* = 9) mOS = 12.3 months (7.7–13.3), *P* = 0.70; (**b**) Type of PE: anterior (*n* = 6) mOS = 12.2 months (5.9-19.7) versus total (*n* = 22) mOS = 11.5 months (5-17.1) versus posterior (*n* = 4) 12.3 months (6.7-21.8), *P* = 0.88; (**c**) Histopathological type of the tumor: squamous cell carcinoma (*n* = 17) mOS = 20.5 months (4.9 to 33.1) versus adenocarcinoma mOS = 10.7 months (5.9 to 13.3), *P* = 0.01; (**d**) presence of vaginal fistula: absent (*n* = 28) mOS = 11.5 months (4.7-15.6) versus present (*n* = 4) mOS—not reached (16.055.6); (**e**) the need of repeated surgery within 30 days: reoperation within 30 days (*n* = 6) mOS = 4.5 months (3.2-10.7) versus no reoperation within 30 days (*n* = 26) mOS = 13.3 months (5.9-19.7), *P* = 0.02; (**f**) the presence of severe adverse events: severe adverse events reported (*n* = 11) mOS = 10.4 months (4.1-11.3) versus no severe adverse events (*n* = 21) mOS = 19.3 months (8.8-25.9), *P* = 0.03; (**g**) specimen margins status: positive margins (*n* = 5) mOS = 7.7 months (6.6-16.5) versus negative margins (*n* = 27) mOS = 12.3 months (4.7-16.6), *P* = 0.09; (**h**) lymph node metastases: absent (26) mOS = 12.3 (4.3-19.5), *P* = 0.55 versus present (6) mOS 11.5 months (6.2-13.1), *P* = 0.55; (**i**) time to recurrence: 0-12 months (*n* = 10) mOS = 13.3 months (7.7-19.3) versus 12-36 months (*n* = 14) mOS = 16.4 months (4.7-17.5) versus above 36 months (*n* = 8) mOS = 10.6 months (6.6-15.6), *P* = 0.31; (**j**) history of hysterectomy during initial treatment: hysterectomy performed (*n* = 3) mOS = 7.4 months (minimal and maximal value: 1.1 and 78.7) versus no hysterectomy (*n* = 29), mOS = 12.3 months (4.8-18.0), *P* = 0.78. Survival analysis of cervical cancer patients who underwent primary PE according to (**k**) presence of vaginal fistula: absent (*n* = 7) mOS = 12.9 months (8.2-16.4) versus present (*n* = 5) mOS = 22.5 months (9.4-62.9), *P* = 0.09; (**l**) histopathological type of cervical cancer: squamous cell carcinoma (*n* = 4) mOS = 47.6 months (8.0-70.9) versus adenocarcinoma (*n* = 8) mOS = 13.5 months (10.1-19.8); *P* = 0.33; (**m**) specimen margins status: positive margins (*n* = 2) mOS = 12.7 months (minimal and maximal value: 12.9 and 14.1) versus negative margins (*n* = 10) mOS = 13.5 months (8.2-30.5), *P* = 0.77; (**n**) lymph node metastases: absent (*n* = 8) mOS = 17.2 months (11.2-22.4) versus present (*n* = 4) mOS = 11.9 months (7.0-24.4); (**o**) severe adverse events: severe adverse events (*n* = 3) mOS = 19.1 months (minimal and maximal value: 7.4 and 86.7) versus no severe adverse events (*n* = 9), mOS = 12.7 months (9.3-18.5)

We observed no differences in patient survival correlating with the duration of remission (time to recurrence) following initial treatment (*P* = 0.31). Moreover, a history of hysterectomy during initial treatment was not linked to patient OS (*P* = 0.78). Table 2 presents detailed data on the survival of patients treated with PE due to cervical cancer recurrence. The survival curves of patients treated with PE due to cervical cancer recurrence are presented in the Fig. 1a-j.

**Table 2:** Survival analysis of cervical cancer patients treated with pelvic exenteration due to cancer recurrence

Pelvic exenteration in cervical cancer recurrence			
	Median (months)	IQR	*P* value
Initial FIGO stage of the disease			
IB, IIA, and IIB (*n =* 13)	11.4	5.5-41.4	0.70
IIIA and IIIB (*n =* 10)	16.4	3.8-17.1	
IVA and IVB (*n =* 9)	12.3	7.7-13.3	
Type of pelvic exenteration			
Anterior (*n =* 6)	12.2	5.9-19.7	0.88
Total (*n =* 22)	11.5	5-17.1	
Posterior (*n =* 4)	12.3	6.7-21.8	
Survival in relationship to histopathological type of cervical cancer			
Squamous cell carcinoma (*n =* 17)	20.5	4.9-33.1	0.01
Adenocarcinoma (*n =* 14)	10.7	5.9-13.3	
Vaginal fistula			
Absent (*n =* 28)	11.5	4.7-15.6	0.02
Present (*n* = 4)	Not reached	16.0-55.6	
The need of repeated surgery			
Reoperation within 30 days (*n =* 6)	4.5	3.2-10.7	0.02
No reoperation within 30 days (*n =* 26)	13.3	5.9-19.7	
Adverse events			
Severe adverse events (*n =* 11)	10.4	4.1-11.3	0.03
No severe adverse events (*n =* 21)	19.3	8.8-25.9	
Specimen margins status			
Positive margins (*n =* 5)	7.7	6.6-16.5	0.09
Negative margins (*n =* 27)	12.3	4.7-16.6	
Lymph node metastases			
Absent (*n =* 26)	12.3	4.3-19.5	0.55
Present (*n =* 6)	11.5	6.2-13.1	
Time to recurrence			
0-12 months (*n =* 10)	13.3	7.7-19.3	0.31
12-36 months (*n =* 14)	16.4	4.7-17.5	
Above 36 months (*n =* 8)	10.6	6.6-15.6	
History of hysterectomy during initial treatment			
Hysterectomy performed (*n =* 3)	7.4	1.1-78.7*	0.78
No hysterectomy (*n =* 29)	12.3	4.8-18.0	

*IQR* interquartile range, corresponds to 25th-75th percentiles

*Minimal and maximal value

In the multivariate survival analysis that considers the histopathological type of the tumor, the presence of fistula, the occurrence of severe adverse events, and the need for reoperation, only histopathological type and reoperation remained independent predictors of patient survival (squamous cell carcinoma positive and reoperation negative, *P* = 0.005).

### The survival analysis of cervical cancer patients treated with primary pelvic exenteration

The median OS of patients treated with primary PE was 14.1 months (IQR: 9.1-22.4). However, when we excluded those patients treated for palliative indications (4 patients with massive bleeding treated in emergency conditions, one patient with ileus, and one patient with metastatic disease), the median OS of patients treated with curative intention improved to 38.8 months (IQR: 9.1-55.0 patients). Only one woman from the patients treated with primary pelvic exenteration is still alive (66 months from the surgery). She is in good condition, without signs of cancer recurrence.

We observed a trend toward improved survival in those patients diagnosed with vaginal fistula compared to those patients without vaginal fistulas; however, the difference in patient survival was not statistically significant (*P* = 0.09). Three patients (25%) experienced one or more serious adverse events. We observed no difference in patient survival correlating with the histopathological type of the tumor (*P* = 0.26), specimen margin status (*P* = 0.77), lymph node metastases (*P* = 0.79), or severe adverse events (*P* = 0.33). Additionally, there were no patients in the analyzed group with a history of hysterectomy who underwent reoperation. Table 3 presents the details of the survival analysis of the group of cervical cancer patients treated with primary PE. The survival curves of patients treated with primary PE are presented in the Fig. 1k-o.

**Table 3:** Survival analysis of cervical cancer patients who underwent primary pelvic exenteration

Primary pelvic exenteration in advanced cervical cancer			
	Median (months)	IQR	*P* value
Squamous cell carcinoma (*n =* 4)	47.6	8.0-70.9	0.26
Adenocarcinoma (*n =* 8)	13.5	10.1-19.8	
Vaginal fistula			
Absent (*n =* 7)	12.9	8.2-16.4	0.09
Present (*n =* 5)	22.5	9.4-62.9	
Severe adverse events			
Severe adverse events (*n =* 3)	19.1	7.4-86.7*	0.33
No severe adverse events (*n =* 9)	12.7	9.3-18.5	
Specimen margins status			
Positive margins (*n =* 2)	12.7	12.9-14.1*	0.77
Negative margins (*n =* 10)	13.5	8.2-30.5	
Lymph node metastases			
Absent (*n =* 8)	17.2	11.2-22.4	0.79
Present (*n =* 4)	11.9	7.0-24.4	

*IQR* interquartile range, corresponds to 25th-75th percentiles

*Minimal and maximal value

## Discussion

Over the last decade, the number of pelvic exenteration procedures performed worldwide has increased significantly. However, those types of surgical procedures may still be associated with high rates of perioperative morbidity (> 50%) [2, 11, 12]. Grade 3, 4, and 5 complications were observed in about 60% of all cases [11], and 10% of patients required surgical reintervention [10]. The most common postoperative complications following pelvic exenteration were hemorrhage (31.8% of cases); ileus (25.8%); wound complications (21.3%); and respiratory failure (16.1%). Other complications included sepsis, thromboembolism, cardiac failure, shock, fistula, and abscess [13]. In a study by Jalloul et al., the types of complications most frequently seen were wound dehiscence (about 55% of cases), urostomy complication, and abscess [11]. In our study, we observed a similar profile of postoperative complications; the most frequently seen were wound complications, and as many as 19% treated due to cancer recurrence required surgical reintervention. In our group of patients, we found a correlation between the need for reoperation and a worse prognosis. Furthermore, the presence of severe adverse events was associated with poor long-term outcomes.

The median perioperative (within 30 days following the procedure) mortality rate for pelvic exenteration is approximately 2% [10]. In the study by Jalloul, the perioperative morbidity rates were as high as 60–95% and the mortality rate was as high as 5% [11]. By contrast, Matsuo et al. saw a reduction in the mortality rate over the last decade from 4.0-7.2% of cases to 1.9–2.3%, as recorded in recent reports [13]. While we did not observe any perioperative mortality in our study, one patient did die on the 56th postoperative day of the hospital stay.

The long-term outcomes in patients treated with exenteration depend on the final surgical clearance. One of the most important factors influencing survival benefit is the achievement of an R0 resection. Whereas the dimensions of the pelvic tumor do not have a significant impact on survival [3], the way in which the border of the healthy tissue margin in the radiated field is determined during resection remains a key factor in establishing survival benefit. When resection was complete, the 3-year overall survival rate reached a median of about 50% in some selected cases, and in cervical cancer cases, it was as high as 73% [10]. Thus, the achievement of R0 resection remains the most important determinant of survival benefit [4–6, 10]. De Gregorio observed a 34.4% 5-year survival rate when R0 was achieved [2] while Li et al. showed that the positive margin of incision was an independent risk factor for poorer overall survival [7]. R1 resection cases were associated with a significantly worse prognosis with a median overall survival time of 10.4 months [3]. In our research, we found significantly longer OS in the group of R0 patients compared with the R1 group; however, the difference did not reach the level of significance. Most likely, the noted difference was due to the small number of R1 patients in our study.

De Georgio et al. found no differences in OS corresponding to the type of histology between patients with squamous cell cancer versus those with adenocarcinoma versus those with other types of carcinomas [2]. Similarly, in a study by Baclabas et al., the histopathological type of the primary tumor did not correlate with improved long-term outcomes [3]. Contrary to these observations, our study found that cases of adenocarcinoma had a worse prognosis than those involving the squamous type of tumor. This result remains the significant prognostic factor in the multivariate analysis. Furthermore, in our study, we had a comparable number of patients with SCC cancers and with cervical adenocarcinomas. By contrast, De Georgio et al. [2] included a small number of cervical adenocarcinoma patients (7) in their study, and Baclabas et al. [3] included patients with pelvic malignancies of various origin, such as rectal and ovarian cancers.

Exenteration may prove therapeutic for more than half of women with node-negative cervical cancer. The stage of the disease, health insurance status, lymph node status, and surgical margins are also independently associated with differential OS after exenteration [14], as are lymphovascular space invasion (LVSI), metastasis to mesorectal lymph nodes, tumor-free margins, and disease-free survival [7]. In a study by De Georgio et al., patients without LVSI had significantly better overall survival [2]. In univariate analysis, LVSI, recurrence or persistent disease, and undergoing a procedure for urinary diversion constituted risk factors for a worse prognosis [3]. In our study, we found no relationship between shorter OS and lymph node involvement. Similar results were obtained in a study by Schmidt et al. that included the largest group of patients treated with pelvic exenteration due to cervical cancer [15].

It has long been recognized that experienced surgeons working in high-volume hospitals offer a superior level of surgical technique associated with a lower rate of surgical complications, decreased operative mortality, and better long-term outcomes. However, using a logistic regression model to examine the potential relationship between surgical experience (assessed by the number of exenteration procedures the surgeon has performed) and long-term outcomes, Jalloul et al. analyzed 167 pelvic exenteration procedures performed by 19 surgeons and were able to demonstrate that the surgeon’s experience did not impact the postoperative complication rate [11]. Although surgical experience was associated with fewer intraoperative transfusions and shorter hospital stays, it was not found to impact patient OS or the rate of postoperative complications [11]. Nevertheless, surgical experience is essential if the tumor nest is to be properly distinguished from radiation-induced fibrosis during the procedure.

Although pelvic exenteration is a combined surgical procedure that requires long operating hours and extended hospitalization and is often correlated with a high risk of excessive complications [16], the survival benefit justifies the application of this procedure in everyday practice. The 5-year overall survival rates following pelvic exenteration due to recurrent cervical cancer have been reported as 32–47% [13]. In our study, we observed a lower rate of long-term survival. However, in a selected group of patients, such as the group of SCC patients, as many as 1/3 of the patients lived longer than 3 years, and 1/4 survived more than 4.5 years. However, survival following PE due to cervical cancer recurrence is influenced by multiple factors. Interestingly, we have observed improved survival in patients whose cancer recurrence was accompanied by the formation of vaginal fistula. To the best of our knowledge, this is the first observation of improved OS associated with the formation of vaginal fistula. It is difficult to explain this finding; however, it is possible that local bacterial infection may correlate with better cancer control [17]. Furthermore, as PE is a very extensive and high-risk procedure, the symptoms related to vaginal fistula may result in quicker decision-making about qualification for PE.

The main limitation of our study is the small number of cases included; however, this limitation is typical of studies on PE. On the other hand, the chief advantage of our study is that it comprises a homogenous group of patients. We focused on only cervical cancer patients and analyzed the results of primary PE and PE for cancer recurrence separately. To increase the overall number of patients, many previous studies have included cases of PE for other types of pelvic malignancies (for example, rectal and ovarian cancers). This practice may be acceptable for reporting early surgical outcomes, but not for analyzing long-term prognostic factors [3, 11]. In our study, we found a correlation between tumor histopathological type and the presence of vaginal fistula with long-term outcomes in cervical cancer patients following PE for cancer recurrence. These are novel findings that require further research to be properly evaluated.

Primary treatment with PE may be an option for selected patients with locally advanced cervical cancer. A group of such patients might include women who present with massive bleeding that cannot be stopped using brachytherapy, those with large tumors for which radical radiotherapy cannot be applied, or those presenting with vaginal fistulas where reconstructive surgery may result in the resolution of bothersome symptoms [15, 18]. The administration of chemotherapy combined with radiation treatment significantly improved the prognosis of patients with locally advanced cervical cancer. However, the improvement in long-term outcomes seems to be more pronounced for patients with stage-IB-IIB cancers compared to those with stage III and IVA cancers [19]. The prognosis for stage IVA cervical cancer patients is still poor, ranging from 32-45% for 3-year OS [20, 21]. Although there has been no prospective randomized trial comparing primary PE and chemoradiation, the long-term outcomes seem to be comparable or better for patients treated with primary PE. Schmidt et al. have reported the OS rates of patients treated with primary PE due to cervical cancer as 41% at 5 years and 37% at 10 years [15]. Similarly, in a study by Chiantera et al., the 5-year OS rate for primary PE patients with cervical cancer was 48% [22]. Although our study included a small group of only 6 patients who were treated with primary PE with curative intention, the results confirmed that primary PE may be an option for selected patients with locally advanced cervical cancer.

## Conclusion

Our results indicate that pelvic exenteration seems to allow for improved long-term outcomes in patients with squamous cell cervical cancer recurrence and vaginal fistula. On the other hand, a poor prognosis correlates with early postoperative morbidity and reoperation, indicating the need for careful patient selection as well as surgical meticulousness and precision.

## Data Availability

Raw data may be requested from the authors with the permission of the institution.
